# Relevance and prognostic ability of Twist, Slug and tumor spread through air spaces in lung adenocarcinoma

**DOI:** 10.1002/cam4.2858

**Published:** 2020-01-22

**Authors:** Ao Liu, Xiao Sun, Jin Xu, Yunpeng Xuan, Yandong Zhao, Tong Qiu, Feng Hou, Yi Qin, Yuanyong Wang, Tong Lu, Yang Wo, Yujun Li, Xiaoming Xing, Wenjie Jiao

**Affiliations:** ^1^ Department of Thoracic Surgery Affiliated Hospital of Qingdao University Qingdao China; ^2^ Department of Pathology Affiliated Hospital of Qingdao University Qingdao China

**Keywords:** lung adenocarcinoma, Slug, tumor spread through air spaces, Twist

## Abstract

**Background:**

Tumor spread through air spaces (STAS) is a novel pathologic characteristic in lung adenocarcinomas that indicates invasive tumor behavior. We aimed to explore the relationship between Twist, Slug and STAS in lung adenocarcinoma and to investigate the potential relationship between epithelial‐mesenchymal transition (EMT) and STAS.

**Materials and methods:**

Our study retrospectively analyzed 115 patients with resected lung adenocarcinomas to evaluate the relationship between Twist, Slug and STAS. STAS was diagnosed using hematoxylin‐eosin (H&E) staining. Immunohistochemistry was used to evaluate the expression levels of Slug and Twist.

**Results:**

In this study, 56 (48.7%) patients had STAS, 40 (34.8%) patients had Slug overexpression, and 28 (24.3%) patients had Twist overexpression. Patients with either STAS or Slug and Twist overexpression experienced poor recurrence‐free survival (RFS) and overall survival (OS). There were significant associations between Twist overexpression, Slug overexpression and the presence of STAS. The logistic model further revealed that pathological stage, Twist overexpression and Slug overexpression were independent risk factors for STAS. A multivariate analysis that contained Twist, Slug, pathologic stage and STAS, showed that pathologic stage and STAS were independent prognostic factors for poor RFS and OS. Another multivariate model that contained Twist, Slug and pathologic stage, showed that pathologic stage, Twist overexpression and Slug overexpression were independent risk factors for poor RFS and OS. In the cohort with STAS, the multivariate analysis showed that pathologic stage and Twist overexpression were independent risk factors for poor survival. The subgroup analysis showed that patients with both Slug overexpression and Twist overexpression with STAS received a poor prognosis.

**Conclusions:**

STAS, Slug and Twist were correlated with poor RFS and OS in resected lung adenocarcinomas. Additionally, STAS was correlated with the overexpression of Twist and Slug, which could potentially provide information on the mechanism of STAS.

## Graphical abstract

In this study, we retrospectively analyzed 115 patients with resected lung adenocarcinomas, aimed to explore the relationship between Twist, Slug and STAS in lung adenocarcinoma and to investigate the potential relationship between epithelial‐mesenchymal transition (EMT) and STAS. We found that STAS, Slug and Twist were correlated with poor RFS and OS in resected lung adenocarcinomas. Additionally, STAS was correlated with the overexpression of Twist and Slug, which could potentially provide information on the mechanism of STAS.

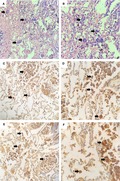

## 1 INTRODUCTION

Spread through air spaces (STAS) is a new pathologic feature of tumor invasion.^1^ It has been reported that cancer cells beyond the edge of the tumor invaded into the alveolar spaces in the lung surrounding parenchyma shown as single cells, micropapillary patterns or solid nests.^2^ Previous studies have reported that STAS was significantly correlated with poor overall survival (OS) and recurrence‐free survival (RFS) in patients with surgically resected lung cancer.^2^, ^3^, ^4^, ^5^, ^6^ STAS was associated with invasive characteristics, such as lymphovascular invasion, visceral pleura invasion and invasive patterns.^2^, ^3^, ^5^, ^6^, ^7^, ^8^, ^9^, ^10^ It was reported that metastasis‐associated protein 1 (MTA1) might provide potential information on the mechanism of STAS.^11^ However, further studies are still needed to identify the molecular characteristics and mechanisms underlying STAS.

The adhesion protein E‐cadherin is one of the hallmarks of epithelial morphogenesis. Tumor cells cannot maintain tissue integrity through the formation of adhesion junctions since losing E‐cadherin, appears as loose tumor cells, which easily escape out of the primary tumor. The decreased expression of cell adhesion molecules, such as E‐cadherin, is one of the main characteristics in the activation of epithelial‐mesenchymal transition (EMT). EMT is considered a major driver of tumor progression from initiation to metastasis.^12^, ^13^ EMT is a highly conserved cellular program that plays key roles in promoting tumor invasion and metastasis. In EMT, polarized epithelial cells lose their epithelial characteristics, intercellular adhesion complexes and cytoskeletal‐specific architecture, and convert into a motile mesenchymal type of cell with polarity, invasive ability and metastatic capacity.^12^, ^13^, ^14^ E‐cadherin expression is downregulated during the acquisition of metastatic potential at the later stages of epithelial tumor progression.^15^, ^16^ Some molecules, including Twist and Slug, have been found to act as E‐cadherin transcription factors that induce EMT.

In view of the association between the migration and invasion of cancer cells with EMT, we hypothesized that STAS regulatory molecules might be associated with EMT. Thus, we investigated the relationship between Twist and Slug with STAS in lung adenocarcinoma and aimed to investigate the potential connection between STAS and EMT.

## 2 MATERIALS AND METHODS

### 2.1 Patients

In this study, we reviewed patients with resected lung adenocarcinoma who were diagnosed between January 2010 and December 2010 at our institution. This retrospective study had been approved by the Institutional Review Board at the Affiliated Hospital of Qingdao University (IRB#QYFYKY 2018‐10‐11‐2). This study followed the Helsinki Declaration. This was a retrospective study, so informed consent was not needed by the Institutional Review Board. The exclusion criteria of this study were as follows: (a) patients with multiple nodules; (b) patients with variants of adenocarcinoma, even with other components, including neuroendocrine or squamous differentiation; and (c) patients who were lost to follow‐up. Patients were included when the following inclusion criteria were met: (a) patients with a single primary tumor; and (b) patients with a confirmed M0 stage. In total, we reviewed 115 patients with resected lung adenocarcinoma in this study. The end follow‐up date was 30 December 2018.

### 2.2 Histopathological examination of STAS

H&E‐stained tumor slides selected in this study were evaluated by two experienced pathologists who were blinded to patient clinical outcomes. Any possible differences were resolved by a consensus after a discussion. Pathologic stage was based on the TNM stage (8th edition). The definition of STAS was based on the 2015 WHO classification. STAS was defined as when cancer cells were located within the alveolar space beyond the edge of the tumor in the lung surrounding the parenchyma accompanied by micropapillary patterns, solid nests and single tumor cells.^2^ First, the boundary of the primary tumor with a smooth surface was confirmed under low‐power microscopy. Then, we identified STAS as when tumor cells or the tumor cell mass was isolated from the primary tumor and the presence of normal alveolar structures beyond the boundary of the primary tumor. STAS was distinguished and confirmed with high‐power field microscopy. Moreover, to discriminate STAS from artificially detached tumor cells or pulmonary loose tumor tissue fragments arising from tumor dissection, a clear vision of a completely normal alveolar structure instead of a broken tissue structure is necessary.

### 2.3 Immunohistochemistry using tissue microarrays

Immunohistochemistry was carried out via two steps. Briefly, first, the paraffin sections were deparaffinized and rehydrated. Following microwave antigen retrieval, we blocked endogenous peroxidase activity by incubating slides in 0.3% H_2_O_2_ and blocked non‐specific binding sites with 10% goat serum for 1 hour. Then, the sections were rinsed and incubated with the anti‐Twist (ab50887, Abcam; diluted 1:100) or anti‐Slug (ab27568, Abcam; diluted 1:100) antibody overnight at 4°C. Then, the Diaminobenzidine Horseradish Peroxidase Color Development Kit (Beyotime, China) was used for color development. Ultimately, the sections were counterstained in hematoxylin and mounted.

Immunohistochemical scores based on the distribution and intensity of staining were used to grade the immunoreactive level of Twist and Slug.^17^ The staining distribution indicates the percentage of positive cells in each core, with scores of 0 (0%), 1 (1%‐50%), and 2 (>50%). The staining intensity was scored as 0 (no expression), 1 (mild expression), 2 (intermediate expression), and 3 (strong expression). Overall, the distribution and intensity scores were summed into a total score (0 to 5) for each patient. Immunostaining scores were dichotomized into low and high. Only a total immunohistochemical score ≥4 was regarded as a positive result.

### 2.4 Statistical analyses

The relationship between STAS and clinicopathologic factors was calculated by the chi‐square test and Student’s t test. A logistic regression model was used to assess the association between patient characteristics and STAS. Univariate Cox regression models were preformed to calculate the prognostic factors. Furthermore, a multivariate Cox regression model was performed to analyse independent predictors. Kaplan‐Meier analyses and log‐rank test were performed to assess RFS and OS. Statistical analyses were performed with IBM SPSS Statistics version 25.0 (IBM Corp.). A *P* value less than .05 was considered statistically significant.

## 3 RESULTS

### 3.1 Patient characteristics

We examined 115 patients who underwent lobectomy in this study. Their detailed clinicopathological characteristics are summarized in Table 1. The mean age of the patients at diagnosis was 60.36 years. Among 115 patients, 27 (23.4%) had pathologic T1 stage disease (T1a stage: 4.3%, T1b stage: 13.9%, T1c stage: 5.2%), 88 (76.6%) had pathologic T2 stage disease (T2a stage: 60.9%, T2b stage: 15.7%), 35 (30.4%) lymph node metastasis (N1 stage: 7.8%, N2 stage: 22.6%), and 82 (24.3%) had the presence of visceral pleural invasion (VPI). All patients were at the M0 stage. During the follow‐up, 61 (53.0%) patients experienced recurrence, while 64 (55.7%) patients died. The mean follow‐up time of the analysis at the endpoint was 76.61 months.

**Table 1:** Clinicopathologic characteristics according to STAS, Twist and Slug in patients with lung adenocarcinoma

Characteristic	All patients (n = 115)	STAS			Slug expression			Twist expression		
		Absence (n = 59)	Presence (n = 56)	*P* Value	Low (n = 75)	High (n = 40)	*P* Value	Low (n = 87)	High (n = 28)	*P* Value
	Number (%)	Number (%)	Number (%)		Number (%)	Number (%)		Number (%)	Number (%)	
Gender										
Male	54 (47.0)	27 (50.0)	27 (50.0)	.792	36 (66.7)	18 (33.3)	.759	43 (79.6)	11 (20.4)	.350
Female	61 (53.0)	32 (52.5)	29 (47.5)		39 (63.9)	22 (36.1)		44 (72.1)	17 (27.9)	
Age										
≤65	82 (71.3)	44 (53.7)	38 (46.3)	.426	52 (63.4)	30 (36.6)	.522	60 (73.2)	22 (26.8)	.328
>65	33 (28.7)	15 (45.5)	18 (54.5)		23 (69.7)	10 (30.3)		27 (81.8）	6 (18.2)	
Smoking history										
Never	73 (63.5)	40 (54.8)	33 (45.2)	.324	48 (65.8)	25 (34.2)	.874	54 (74.0)	19 (26.0)	.580
Former/current	42 (36.5)	19 (45.2)	23 (54.8)		27 (64.3)	15 (35.7)		33 (78.6)	9 (21.4)	
Post‐operative hospitalization days										
Mean ± SD	9.19 ± 2.665	8.81 ± 2.345	9.59 ± 2.934	.119	8.79 ± 2.332	9.95 ± 3.088	.025	9.32 ± 2.764	8.79 ± 2.331	.357
Range	5‐17	5‐17	5‐17		5‐17	7‐17		5‐17	5‐17	
Tumor location										
RUL	48 (41.7)	24 (50.0)	24 (50.0)	.084	30 (62.5)	18 (37.5)	.726	39 (81.2)	9 (18.8)	.434
RML	8 (7.0)	4 (50.0)	4 (50.0)		4 (50.0)	4 (50.0)		4 (50.0)	4 (50.0)	
RLL	18 (15.7)	6 (33.3)	12 (66.7)		13 (72.2)	5 (27.8)		13 (72.2)	5 (27.8)	
LUL	25 (21.7)	12 (48.0)	13 (52.0)		16 (64.0)	9 (36.0)		19 (76.0)	6 (24.0)	
LLL	16 (13.9)	13 (81.2)	3 (18.8)		12 (75.0)	4 (25.0)		12 (75.0)	4 (25.0)	
Tumor size (cm)										
0‐3	69 (60.0)	33 (47.8)	36 (52.2)	.361	48 (69.6)	21 (30.4)	.231	48 (69.6)	21 (30.4)	.062
3‐5	46 (40.0)	26 (56.5)	20 (43.5)		27 (58.7)	19 (41.3)		39 (84.8)	7 (15.2)	
Mean ± SD	3.003 ± 1.237	3.127 ± 1.216	2.871 ± 1.256		2.989 ± 1.272	3.028 ± 1.184		3.161 ± 1.235	2.511 ± 1.126	
Pathologic differentiation										
Low	35 (30.4)	16 (45.7)	19 (54.3)	.178	25 (71.4)	10 (28.6)	.033	28 (80.0)	7 (20.0)	.698
Median	60 (52.2)	29 (48.3)	31 (51.7)		33 (55.0)	27 (45.0)		45 (75.5)	15 (25)	
High	20 (17.4)	14 (70.0)	6 (30.0)		17 (85.0)	3 (15.0)		14 (70.0)	6 (30.0)	
Lymph node metastasis										
No	80 (69.6)	48 (60.0)	32 (40.0)	.005	54 (67.5)	26 (32.5)	.437	62 (77.5)	18 (22.5)	.485
Yes	35 (30.4)	11 (31.4)	24 (68.6)		21 (60.0)	14 (40.0)		25 (71.4)	10 (28.6)	
Pathologic T stage										
T1	27 (23.5)	17 (63.0)	10 (37.0)	.166	23 (85.2)	4 (14.8)	.013	20 (74.1)	7 (25.9)	.827
T2	88 (76.5)	42 (47.7)	46 (52.3)		52 (59.1)	36 (40.9)		67 (76.1)	21 (23.9)	
Pathologic N stage										
N0	80 (69.6)	48 (60.0)	32 (40.0)	.012	54 (67.5)	26 (32.5)	.701	62 (77.5)	18 (22.5)	.686
N1	9 (7.8)	4 (44.4)	5 (55.6)		5 (55.6)	4 (44.4)		7 (77.8)	2 (22.2)	
N2	26 (22.6)	7 (26.9)	19 (73.1)		16 (61.5)	10 (38.5)		18 (69.2)	8 (30.8)	
Pathologic stage										
Stage I	69 (60.0)	42 (60.9)	27 (39.1)	.013	47 (68.1)	22 (31.9)	.722	51 (73.9)	18 (26.1)	.231
Stage II	20 (17.4)	10 (50.0)	10 (50.0)		12 (60.0)	8 (40.0)		18 (90.0)	2 (10.0)	
Stage III	26 (22.6)	7 (26.9)	19 (73.1)		16 (61.5)	10 (38.5)		18 (69.2)	8 (30.8)	
VPI										
Absence	33 (28.7)	23 (69.7)	10 (30.3)	.012	27 (81.8)	6 (18.2)	.018	26 (78.8)	7 (21.2)	.619
Presence	82 (71.3)	36 (43.9)	46 (56.1)		48 (58.5)	34 (41.5)		61 (74.4)	21 (25.6)	
Twist expression										
Low	87 (75.7)	53 (60.9)	34 (39.1)	<.001	60 (69.0)	27 (31.0)	.137	—	—	—
High	28 (24.3)	6 (21.4)	22 (78.6)		15 (53.6)	13 (46.4)		—	—	
Slug expression										
Low	75 (65.2)	45 (60.0)	30 (40.0)	.011	—	—	—	60 (80.0)	15 (20.0)	.137
High	40 (34.8)	14 (35.0)	26 (65.0)		—	—		27 (67.5)	13 (32.5)	

Abbreviations: LLL, left lower lobe; LUL, left upper lobe; RLL, right lower lobe; RML, right middle lobe; RUL, right upper lobe; SD, standard deviation; STAS, spread through air spaces; VPI, visceral pleural invasion.

### 3.2 Association between slug, twist and clinicopathological characteristics

The associations between clinicopathologic factors, the expression of Slug and Twist and the presence of STAS are summarized in Table 1. Among the 115 patients examined in this study, 75 (65.2%) had Slug overexpression and 28 (24.3%) had Twist overexpression. As shown in Table 1, correlations between the expression levels of Slug and Twist and clinicopathological characteristics are listed. There was a significant relationship between Slug overexpression and pathologic differentiation (*P* = .033), pathologic T stage (*P* = .013), and the presence of VPI (*P* = .018). Slug overexpression showed a potential correlative trend with lymph node metastasis (32.5% vs 40.0%). On the other hand, Twist overexpression was not significantly associated with a high pathologic stage. However, there could be potential correlations between Twist overexpression and high pathologic differentiation (20.0% vs 25.0% vs 30.0%), increased lymph node metastasis (22.5% vs 28.6%), and the presence of VPI (21.2% vs 25.6%). Regarding the overexpression of Slug and Twist, increased Slug overexpression could be associated with increased Twist overexpression (31.0% vs 46.4%, *P* = .137) and vice versa (20.0% vs 32.5%, *P* = .137).

### 3.3 Association between STAS and clinicopathological characteristics

**Figure 1:**
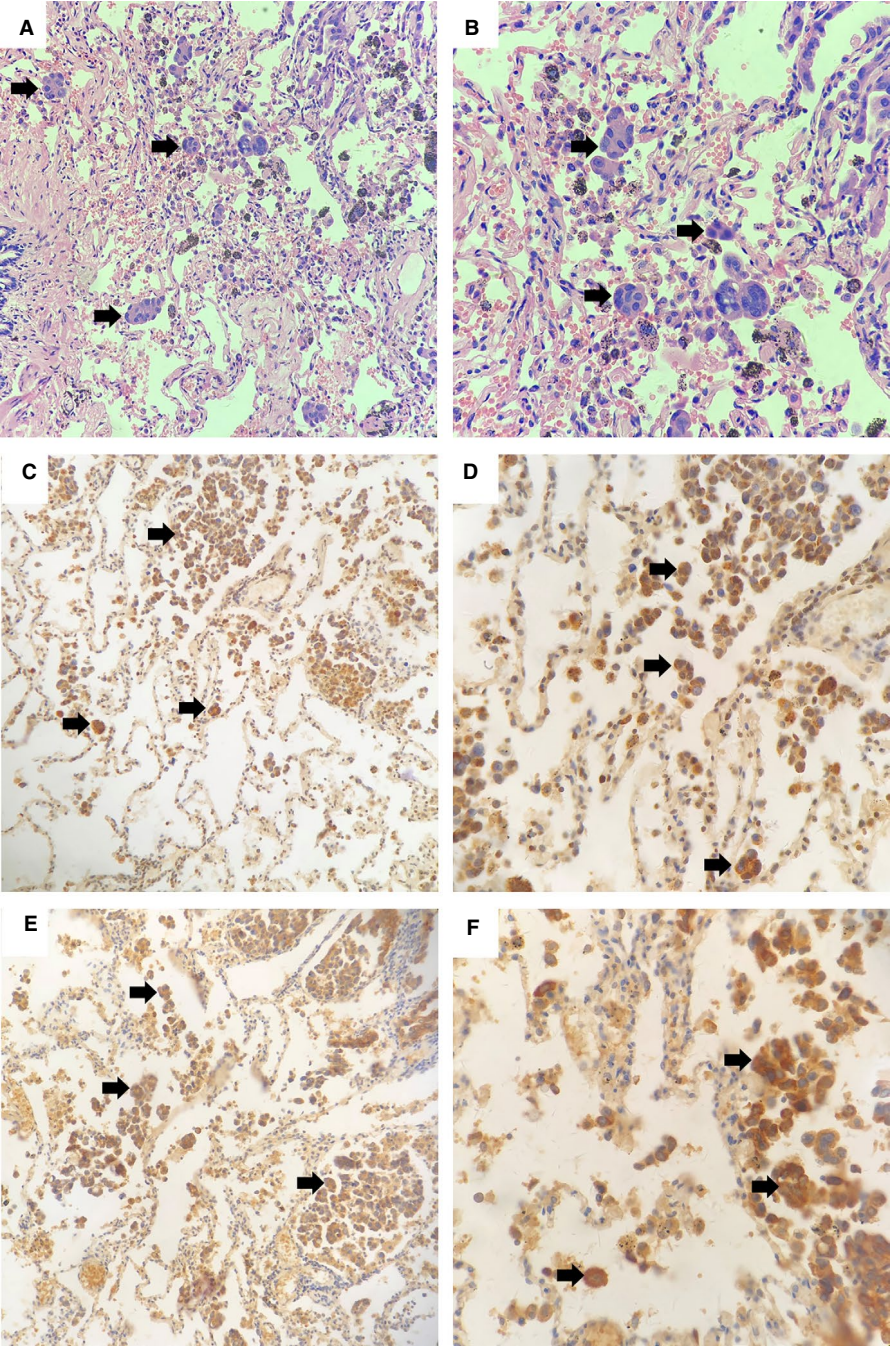
A/B, Representative histopathologic images of spread through air spaces (STAS) in lung adenocarcinomas (arrows). A, magnification ×200, field of a tumor section with STAS. B, magnification ×400, field of a tumor section with STAS. C, Twist expression in lung adenocarcinomas (magnification ×200). D, Twist expression in lung adenocarcinomas (magnification ×400). E, Slug expression in lung adenocarcinomas (magnification ×200). F, Slug expression in lung adenocarcinomas (magnification ×400)

Associations between clinicopathologic characteristics and the presence of STAS are shown in Table 1. STAS was observed in 56 patients (48.7%). Representative microphotographs of STAS are shown in Figure 1. There was a significant association between the presence of STAS and lymph node metastasis (*P* = .005) as well as pathologic N stage (*P* = .012), pathologic stage (*P* = .013), the presence of VPI (*P* = .012), Twist expression (*P* < .001) and Slug expression (*P* = .011).

An additional logistic regression analysis was performed to calculate the association between STAS and pathological factors. The logistic regression analysis contained pathologic stage, Twist and Slug (Table 2). The logistic model further revealed that pathological stage (OR = 2.154, 95% CI = 1.277‐3.636, *P* = .004), Twist overexpression (OR = 6.104, 95% CI = 2.104‐17.711, *P* = .001) and Slug overexpression (OR = 2.471, 95% CI = 1.030‐5.929, *P* = .043) were independent risk factors for STAS.

**Table 2:** Logistic regression analysis of factors associated with STAS in patients with lung adenocarcinoma

Characteristic, factor	Logistic regression analysis STAS		
	OR	95% CI	*P* Value
Pathologic stage (per stage)	2.154	1.277‐3.636	.004
Twist, overexpression	6.104	2.104‐17.711	.001
Slug, overexpression	2.471	1.030‐5.929	.043

Abbreviations: CI, confidence interval; OR, odds ratio; STAS, spread through air spaces.

### 3.4 Survival analysis

The univariate Cox proportional hazards regression analysis was performed to assess the prognostic risk factors for RFS and OS (Table 3). Pathologic N stage (*P* < .001), presence of VPI (*P* = .069 vs *P* = .011), pathologic stage (*P* < .001), presence of STAS (*P* < .001), Twist overexpression (*P* = .037 vs *P* = .010) and Slug overexpression (*P* = .015 vs *P* = .002) were significant prognostic risk factors for RFS and OS. Furthermore, patients with STAS experienced worse RFS or OS than those without STAS (hazard ratio (HR), 3.696; 95% confidence interval (CI), 2.152‐6.348; *P* < .001 vs HR, 4.577; 95% CI, 2.639‐7.937; *P* < .001) in the univariate analysis. Moreover, patients with Slug overexpression also experienced worse RFS or OS than those without Slug overexpression (HR, 1.885; 95% CI, 1.132‐3.140; *P* = .015 vs HR, 2.155; 95% CI, 1.314‐3.532; *P* = .002). Similarly, patients with Twist overexpression also experienced worse RFS or OS than those without Twist overexpression (HR, 1.802; 95% CI, 1.037‐3.131; *P* = .037 vs HR, 2.002; 95% CI, 1.178‐3.404; *P* = .010).

**Table 3:** Univariate and multivariate analyses of RFS and OS in patients with lung adenocarcinoma

Characteristic, factor	Univariate analysis						Multivariate analysis						Multivariate analysis					
	RFS			OS			RFS			OS			RFS			OS		
	HR	95% CI	*P* Value	HR	95% CI	*P* Value	HR	95% CI	*P* Value	HR	95% CI	*P* Value	HR	95% CI	*P* Value	HR	95% CI	*P* Value
Clinical factors																		
Gender, male	0.888	0.537‐1.467	.643	1.057	0.647‐1.728	.824	—	—	—	—	—	—	—	—	—	—	—	—
Age, older, ＞65	1.033	0.595‐1.792	.909	1.261	0.748‐2.126	.385	—	—	—	—	—	—	—	—	—	—	—	—
Smoking history, former/current	1.255	0.750‐2.098	.387	1.174	0.710‐1.940	.533	—	—	—	—	—	—	—	—	—	—	—	—
Tumor location, lower lobe	1.357	0.800‐2.302	.257	0.884	0.512‐1.525	.657	—	—	—	—	—	—	—	—	—	—	—	—
Pathologic factors																		
Tumor size (cm), larger (per cm)	1.144	0.928‐1.412	.208	1.046	0.850‐1.286	.673	—	—	—	—	—	—	—	—	—	—	—	—
Pathologic differentiation	1.334	0.925‐1.925	.123	1.218	0.850‐1.745	.282	—	—	—	—	—	—	—	—	—	—	—	—
Pathologic T stage (per stage)	1.187	0.922‐1.529	.184	1.263	0.976‐1.635	.076	—	—	—	—	—	—	—	—	—	—	—	—
Pathologic T stage, T2 vs T1	1.598	0.849‐3.010	.147	2.135	1.086‐4.197	.028	—	—	—	—	—	—	—	—	—	—	—	—
Pathologic N stage (per stage)	1.822	1.384‐2.400	<.001	1.693	1.293‐2.215	<.001	—	—	—	—	—	—	—	—	—	—	—	—
Lymph node metastasis, yes vs no	2.884	1.727‐4.815	<.001	2.590	1.572‐4.269	<.001	—	—	—	—	—	—	—	—	—	—	—	—
Pathologic stage (per stage)	1.376	1.179‐1.606	<.001	1.351	1.164‐1.568	<.001	1.783	1.313‐2.421	<.001	1.726	1.277‐2.332	<0.001	2.016	1.494‐2.720	<.001	1.897	1.408‐2.556	<.001
Pathologic stage, II vs I	1.673	0.838‐3.341	.145	1.637	0.843‐3.181	.146	—	—	—	—	—	—	—	—	—	—	—	—
Pathologic stage, III (vs I & II)	3.049	1.776‐5.233	<.001	2.586	1.520‐4.401	<.001	—	—	—	—	—	—	—	—	—	—	—	—
VPI, Presence	1.742	0.957‐3.170	.069	2.272	1.211‐4.263	.011	—	—	—	—	—	—	—	—	—	—	—	—
STAS, Presence	3.696	2.152‐6.348	<.001	4.577	2.639‐7.937	<.001	2.638	1.464‐4.754	.001	3.475	1.934‐6.243	<0.001	—	—	—	—	—	—
Twist, overexpression	1.802	1.037‐3.131	.037	2.002	1.178‐3.404	.010	1.684	0.910‐3.116	.097	1.732	0.956‐3.137	0.070	2.343	1.297‐4.232	.005	2.568	1.439‐4.582	.001
Slug, overexpression	1.885	1.132‐3.140	.015	2.155	1.314‐3.532	.002	1.389	0.822‐2.349	.220	1.475	0.889‐2.449	0.133	1.719	1.029‐2.872	.039	1.869	1.135‐3.077	.014

Abbreviations: CI, confidence interval; HR, hazard ratio; HS, histological subtype; OS, overall survival; RFS, recurrence‐free survival; STAS, spread through air spaces; VI, vascular invasion; VPI, visceral pleural invasion.

Additional multivariate models were used to analyze independent predictors (Table 3). One multivariate analysis including pathologic stage, STAS, Twist and Slug showed that pathologic stage (HR, 1.783; 95% CI, 1.313‐2.421; *P* < .001) and the presence of STAS (HR, 2.638; 95% CI, 1.464‐4.754; *P* = .001) were independent prognostic risk factors for poor RFS. The same outcome was shown in the multivariate models of OS (pathologic stage: HR, 1.726; 95% CI, 1.277‐2.332; *P* < .001; presence of STAS: HR, 3.475; 95% CI, 1.934‐6.243; *P* < .001).

Another multivariate model that contained Twist, Slug and pathologic stage was applied. Pathologic stage (HR, 2.016; 95% CI, 1.494‐2.720; *P* < .001), Twist overexpression (HR, 2.343; 95% CI, 1.297‐4.232; *P* = .005), and Slug overexpression (HR, 1.719; 95% CI, 1.029‐2.872; *P* = .039) were identified as independent risk factors for poor RFS. The same outcome was shown for OS (pathologic stage: HR, 1.897; 95% CI, 1.408‐2.556; *P* < .001; Twist overexpression: HR, 2.568; 95% CI, 1.439‐4.582; *P* = .001; Slug overexpression: HR, 1.869; 95% CI, 1.135‐3.077; *P* = .014).

**Figure 2:**
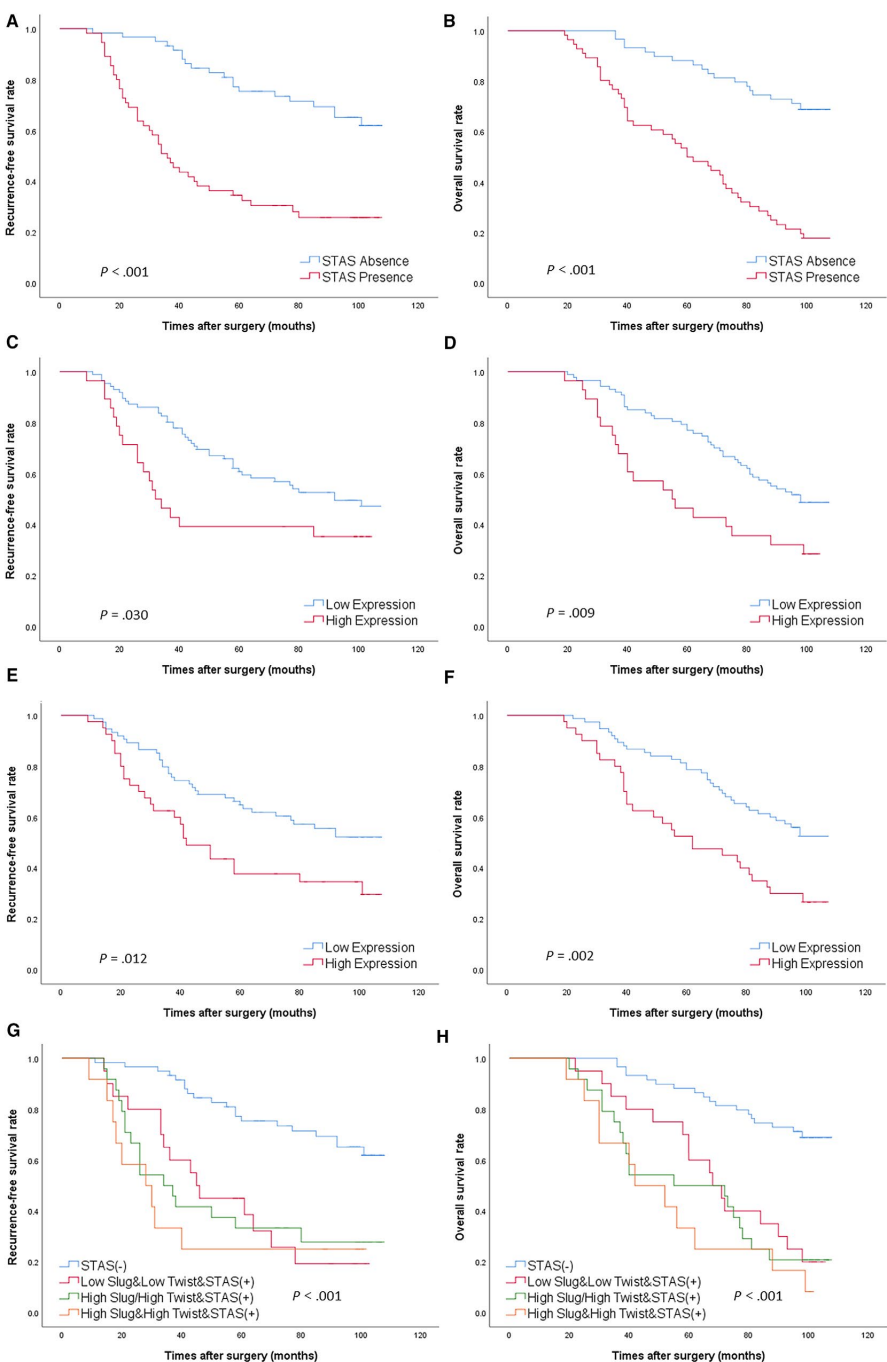
Kaplan‐Meier survival analysis of recurrence‐free survival (RFS) and overall survival (OS) according to spread through air spaces (STAS), Twist and Slug in patients with lung adenocarcinoma. A, RFS in patients with vs without STAS (*P* < .001). B, OS in patients with vs without STAS (*P* < .001). C, RFS in patients with high vs low Twist expression (*P* = .030). D, OS in patients with high vs low Twist expression (*P* = .009). E, RFS in patients with high vs low Slug expression (*P* = .012). F, OS in patients with high vs low Slug expression (*P* = .002). G, RFS in four groups of patients (*P* < .001). H, OS in four groups of patients (*P* < .001)

Kaplan‐Meier analysis was performed to determine survival rates according to the expression of Twist and Slug and the presence of STAS (Figure 2). Patients with STAS experienced significantly poorer RFS and OS than patients without STAS (*P* < .001). The 5‐year RFS rates in patients with and without STAS were 35.7% and 75.3%, respectively, and the 5‐year OS rates were 50.0% and 88.1%, respectively. Similarly, patients with Twist overexpression also experienced significantly poorer RFS and OS than patients with low Twist expression (*P* = .030 vs *P* = .009). Patients with Slug overexpression also experienced poor RFS and OS (*P* = .012 vs *P* = .002).

### 3.5 Survival analysis in the cohort according to Slug, Twist and STAS

All patients were divided into two cohorts according to STAS (Table 4). In the cohort with STAS, the multivariate analysis showed that pathologic stage (HR, 1.356; 95% CI, 1.101‐1.670; *P* = .004) was a significant risk factor for poor RFS. Additionally, Twist overexpression (HR, 1.737; 95% CI, 0.882‐3.422; 0.110) could be acceptable as a risk factor for poor RFS. On the other hand, pathologic stage (HR, 1.519; 95% CI, 1.234‐1.869; *P* < .001) and Twist overexpression (HR, 2.317; 95% CI, 1.203‐4.463; 0.012) were revealed as significant risk factors for poor OS.

**Table 4:** Univariate and multivariate analyses of RFS and OS in lung adenocarcinoma patients with STAS

Characteristic, factor	STAS presence											
	Univariate analysis						Multivariate analysis					
	RFS			OS			RFS			OS		
	HR	95% CI	*P* Value	HR	95% CI	*P* Value	HR	95% CI	*P* Value	HR	95% CI	*P* Value
Pathologic stage (per stage)	1.286	1.056‐1.564	.012	1.387	1.148‐1.676	.001	1.356	1.101‐1.670	.004	1.519	1.234‐1.869	<.001
Twist, overexpression	1.274	0.678‐2.396	.451	1.461	0.810‐2.635	.208	1.737	0.882‐3.422	.110	2.317	1.203‐4.463	.012
Slug, overexpression	1.117	0.604‐2.069	.724	1.214	0.680‐2.167	.512	1.141	0.615‐2.117	.675	1.193	0.666‐2.135	.553

Abbreviations: CI, confidence interval; HR, hazard ratio; RFS, recurrence‐free survival; STAS, spread through air spaces.

Furthermore, all patients were divided into four groups according to Slug, Twist and STAS as follows: Group 1: STAS(−): patients with the absence of STAS regardless of the expression of Slug and Twist; Group 2: Low Slug/Low Twist&STAS(+): patients without Slug and Twist overexpression and with STAS; Group 3: High Slug/High Twist&STAS(+): patients with either Slug or Twist overexpression and with STAS; and Group 4: High Slug&High Twist&STAS(+): patients with both Slug and Twist overexpression and with STAS. Kaplan‐Meier analysis was used to determine survival rates in the four cohorts (Figure 2). RFS and OS became increasingly worse from group 1 to group 4 (*P* < .001).

## 4 DISCUSSION

In this study, we examined the relationship between Twist, Slug and STAS in patients with resected stage I‐III lung adenocarcinoma and aimed to investigate the potential connection between STAS and EMT. To our knowledge, this is the first study to assess the relationship between Slug, Twist and STAS in resected pathologic stage I‐III lung adenocarcinoma.

Regarding the definition of STAS, STAS is a novel pathologic feature in lung cancer that includes three histomorphological characteristics: solid nests, micropapillary patterns and single cells.^2^ STAS is also associated with recurrence and survival in squamous cell carcinoma,^18^, ^19^, ^20^ lung large cell neuroendocrine carcinoma, and lung small cell carcinoma,^21^ even lung pleomorphic carcinoma.^22^ STAS has been reported to be related with an aggressive tumor behavior, such as a larger tumor size,^6^, ^18^, ^23^ a high pathologic stage,^3^, ^4^, ^8^, ^24^, ^25^ lymphatic or vascular invasion,^2^, ^4^, ^6^, ^7^, ^8^, ^10^, ^18^, ^23^, ^25^ pleural invasion,^4^, ^6^, ^7^, ^10^, ^23^, ^24^ tumor budding^19^ and increased micropapillary or solid structures in lung adenocarcinomas.^2^, ^3^, ^5^, ^6^, ^7^, ^8^, ^9^, ^10^ We previously reported that the positive rate of STAS may be different for different invasive patterns and even lymph node metastasis.^10^ Thus, in this study, it was acceptable for the positive rate of STAS to be 48.7% in all patients with stage I‐III lung adenocarcinomas. It was shown that STAS was significantly related to lymph node metastasis, pathologic stage and presence of VPI. Additionally, patients with STAS experienced poor RFS or OS, consistent with previous studies.

Based on current research, STAS has important significance as a reference in patient prognosis. It was reported that MTA1 might provide potential information on the mechanism of STAS.^11^ However, further studies are still needed to identify the molecular characteristics and mechanisms underlying STAS. From a histomorphological perspective, tumor cells or tumor masses migrate from the primary tumor to the surrounding alveolar spaces beyond the edge of the primary tumor. The primary tumor, which is more prone to STAS, is frequently accompanied by loose tumor tissue. In addition, it has been reported that there are fewer intercellular adhesions among poorly differentiated discohesive tumor cells, including poorly differentiated squamous cell carcinomas and micropapillary adenocarcinomas.^26^, ^27^ Interestingly, it has been reported that loose cells in micropapillary carcinoma most likely facilitate anchorage‐independent growth and acquire resistance to apoptosis, which are advantageous for proliferation during lymphatic cancer metastasis.^28^ On the other hand, the presence of frequent STAS‐like and decreasing E‐cadherin expression in ROS1‐rearranged lung cancer can predict lower disease‐free survival.^29^ Additionally, STAS was proven to be associated with micropapillary patterns.^2^, ^3^, ^5^, ^6^, ^7^, ^8^, ^9^, ^10^ Thus, we hypothesized that STAS could be associated with poor intercellular adhesions among tumor cells, by which the tumor cells could spread into the alveolar spaces beyond the edge of the primary tumor. Furthermore, a key molecule that maintains tissue integrity through the formation of adhesion junctions is E‐cadherin. Thus, the loss of E‐cadherin among tumor cells results in the degradation of cell‐cell adhesions and the isolation of malignant cells from the epithelial layer, making it easy for loose tumor cells to escape from the primary tumor.

The role of EMT in promoting metastasis and cancer invasion was recently considered important. EMT is a process whereby epithelial cell layers with a loss of epithelial cell adhesion and cytoskeletal components undergo remodeling of the cytoskeleton. The main characteristics of EMT are the decrease in the expression of cell adhesion molecules such as E‐cadherin, the transformation of a cytokeratin cytoskeleton into a vimentin‐based cytoskeleton, and the morphological characteristics of mesenchymal cells.^15^, ^16^ EMT is an important biological process for epithelial‐derived malignant tumor cells to acquire migration and invasion abilities. Through EMT, epithelial cells lose cell polarity and junctions with the basement membrane and obtain higher migration and invasion, antiapoptotic and extracellular matrix degrading abilities and other mesenchymal phenotypes. Tumor cells with EMT acquire the expression of mesenchymal components and manifest a migratory phenotype. Thus, EMT can facilitate tumor cells to become loose and disseminate from the primary tumors.^12^, ^13^ On the other hand, it has been reported that STAS could be related to tumor budding.^19^ Concerning tumor budding, it has been reported that tumor budding reflects invasive tumor behavior and is an adverse prognostic factor.^30^, ^31^, ^32^, ^33^ Tumor budding is thought to be closely related to EMT because the molecular mechanism that leads to budding may be, in part, the same as that of EMT,^34^ thereby increasing cancer cell migration and invasion.^33^, ^35^, ^36^ It was recently determined that the presence of tumor budding was significantly associated with the downregulation of E‐cadherin and the acquisition of vimentin expression in cancer cells.^30^ In view of the existing research, STAS may be related to EMT. Furthermore, because EMT and STAS are largely restricted to the interface between the malignant epithelium and stromal elements at the invasive margin, an induction of EMT may trigger STAS.

A hallmark of EMT is the loss of E‐cadherin expression, which is consistently observed at sites of EMT during cancer. E‐cadherin transcription factors, such as Twist and Slug, can induce EMT. Twist is an inducer of EMT and is correlated with poor survival.^15^ Twist overexpression has been found among tumor tissues. When Twist expression is significantly inhibited, the metastatic potential of cells can be impaired. Moreover, a previous study showed that Twist overexpression could decrease OS in patients with lung cancer.^37^ On the other hand, Twist overexpression also decreased RFS.^38^ Slug is a member of the zinc finger Snail family. Slug is a significant EMT inducer that has been implicated in the progression of lung cancer.^39^ When overexpressed, Slug abrogates E‐cadherin‐mediated intercellular adhesion and promotes tumor cell invasion.^40^ Slug overexpression was found to be correlated with both poor disease‐free survival and OS in lung cancer.^41^, ^42^, ^43^ In this study, Slug overexpression was significantly related to high pathologic differentiation, a high pathologic T stage, the presence of VPI, and increased lymph node metastasis. Furthermore, Twist overexpression showed a trend with high pathologic differentiation, increased lymph node metastasis, and the presence of VPI. Similar to other studies, we also found that Twist and Slug overexpression was associated with decreased RFS and OS in patients with lung adenocarcinoma according to the survival analysis. Thus, we can conclude that the overexpression of Slug and Twist could be related to invasive tumor behavior, such as STAS.

In our study, STAS was significantly associated with the overexpression of Slug and Twist. An additional logistic model further revealed that pathological stage, Twist overexpression and Slug overexpression were independent risk factors for STAS. It seems that the presence of STAS was related to Twist and Slug overexpression. The univariate Cox analysis showed that pathologic stage, STAS, Twist and Slug were significant risk factors for RFS and OS. One multivariate model that contained pathologic stage, STAS, Twist and Slug showed that the significantly independent prognostic risk factors of RFS and OS were STAS and pathologic stage. Considering the potential connection between STAS, Twist, and Slug, another multivariate model that contained Twist, Slug and pathologic stage (and not STAS) was applied. This multivariate analysis showed that pathologic stage, Twist and Slug were significant independent prognostic factors for RFS and OS. Thus, we inferred that STAS, Twist overexpression and Slug overexpression could influence each other in terms of prognosis. Furthermore, the potential relationship between STAS and the overexpression of Slug and Twist was confirmed in another manner.

To investigate the prognostic impact of Slug or Twist in patients with STAS, all patients were divided into several groups according to Slug, Twist or STAS. In the cohort with STAS, the multivariate analysis showed that pathologic stage and Twist overexpression were independent risk factors for poor survival. Therefore, Twist expression could serve as a stratification factor to evaluate prognosis among patients with the same pathologic stage and STAS. However, Slug overexpression was not identified as an independent risk factor in patients with STAS. This consequence may be related to STAS and Slug. Additionally, there was no correlation between STAS and Slug. Furthermore, we divided all patients into four groups according to Slug, Twist and STAS. RFS and OS became increasingly worse from group 1 to group 4. Patients with both Slug and Twist overexpression and STAS experienced a poor prognosis. Currently, we cannot clearly recognize the roles of Slug and Twist in the molecular mechanism of STAS, and we do not know the molecular mechanism of STAS and related signal transduction pathways. Our research shows that STAS is associated with Slug and Twist. Because Slug and Twist play an important role in the occurrence of EMT, STAS may have a potential relationship with EMT, and the induction of EMT may trigger STAS. Our study provides important reference information for further study of the molecular mechanism of STAS and related signal transduction pathways.

Furthermore, some studies have reported that Twist may be correlated with multidrug resistance, such as vincristine^44^ and paclitaxel,^45^, ^46^ which are chemotherapeutic drugs. Furthermore, in lung adenocarcinoma with EGFR mutations, Slug could be correlated with resistance to gefitinib.^47^ However, we found that STAS was correlated with the overexpression of Twist and Slug. Therefore, STAS may be a novel treatment target by increasing drug responsiveness and sensitivity to improve prognosis.

Our study had several limitations. First, it was a retrospective study of a small sample size from a single institution. Selection bias was ineluctable. Second, we measured the expression levels of Slug and Twist only by immunohistochemistry because the tissue was previously embedded in paraffin. Therefore, it is necessary to further demonstrate the relationship between Twist, Slug and STAS and the molecular mechanism of STAS and related signal transduction pathways in additional multicenter prospective studies with larger samples with additional cell biology methods. Third, the related driver gene mutations and postoperative records of targeted therapy remain unknown.

## 5 CONCLUSIONS

STAS, Slug and Twist were correlated with poor RFS and OS in resected lung adenocarcinomas. Additionally, STAS was correlated with the overexpression of Twist and Slug, which could potentially provide information on the mechanism of STAS.

## CONFLICT OF INTEREST

All authors declare that they have no competing interests.

## Data Availability

Please contact the corresponding author for the original data.
